# Parasitic infection in various stages life of cultured *Acipenser persicus*

**Published:** 2016-03-15

**Authors:** Milad Adel, Reza Safari, Zahra Yaghoubzadeh, Hassan Fazli, Elham Khalili

**Affiliations:** 1 *Department of Aquatic Animal Health and Diseases, Caspian Sea Ecology Research Center, Sari, Iran; *; 2 *DVMGraduate, Faculty of Veterinary Medicine, Shahrekord University, Shahrekord, Iran.*

**Keywords:** *Acipenser persicus*, Iran, Parasite

## Abstract

The present study was conducted to evaluate the status of the parasite fauna in *Acipenser persicus* at different development stages, in order to find prevention protocols for parasitic diseases in this valuable species. For this purpose, sampling from each sex breeder, 10 egg samples, 5-day-old larvae (n = 20), 20-day-old larvae (n = 80) and fingerling of *A. persicus* (n = 60) released in earthen ponds were done. After the bioassay and preparing wet mount from the internal and external organs, identification was done according to the keys. According to the results, no fauna parasites were isolated from egg samples and 5-day-old larvae; but *Trichodina *spp. was isolated from 20-day-old larvae. Also, the same protozoan was isolated from fingerling released in earthen ponds, the mean intensity, prevalence and range of contamination by fingerling were higher with compared to 20-day-old larvae. *Trichodina* sp. and *Diplostomum spathaceum* were isolated from skin and eyes of females, respectively. However, *Trichodina *sp*.* and *Ichthyophthirius multifiliis* were isolated from skin of male breeders. In this study, no parasites were isolated from internal organs of larves and fingerling but four intestinal parasites included: *Cucullanus sphaerocephlaus*, *Anisakis *sp*., Skyrjabinopsilus semiarmatus,* and *Lepto-rhynchoides plagicephalu* were isolated from internal organs of breeder. Based on a wide range of parasitic infection observed in various life stages of *A. persicus*, it seems necessary to consider hygienic and management measures.

## Introduction

*Acipenser persicus* is one of the most important species in the family of Acipenseridae which its meat and caviar are considered economically. This fish is distributed along by Koura River (Azerbaijan) and Sefidrood River (Iran).^1^ During the past two decades, overfishing, loss of habitat for natural reproduction, industrial and domestic pollution and increase of infectious diseases puts the reservoir in alarming rate.^1^ Therefore, artificial culture of *A. persicus* has been increased in aquaculture industry of Iran. In-breeding and rearing sturgeon aquaculture requires comprehensive information about health status and diseases, not only conduce to the promotion of health and quality of sturgeon, but also could have an important role in the production and proliferation of endangered species.^2^

Parasitical pathogens could effect on physiological and biological features and their mechanical damages may also predispose fish to the viral, bacterial and fungal disease and cause severe mortality.^3^ Therefore, identification of infectious pathogenic parasites could be helpful in proliferation and rearing of different sturgeon species, especially *A. persicus.*

So far, more than 60 species of parasites in sturgeons of the Black Sea and the Caspian Sea have been recorded.^4^ The first study in parasitic fauna of sturgeons in Iran, was done by Mokhayer in 1973 which reported *Amphilina pholiacea*.^4^ Subsequently, Niyak *et al.*, Mokhayer, Shenavar Masouleh, Pazooki and Masoumian, Sattari and Mokhayer, Bazari Moghaddam *et* *al*. and Noei investigated the parasitic fauna of different sturgeon species and identified different parasitic species, i.e., *Ichthyophthirius multifiliis, Diplostomum spathaceum,*^5^* Trichodina reticulata,*^6^* Cryptobia acipenseris,*^7^* Polypodium hydriforme,*^7^* Hemogregarina acipenseris,*^7^* Skrjabinopsolus skrjabini, S. acipenseris, Amphilina foliacea, Bothrimonus fallax, Eubothrium acipenserinum,*^8^* Ascarophis ovotrichuria, Cyclozone acipenserina, Cucullanus sphaero-cephalus, Contracaecum *sp*., Anisakis *sp*.,*^8^* Eustrongylides excisus, Leptorhynchoides plagicephalus, Pomphorhynchus laevis, Corynosoma capsicum *and *Ligula intestinalis*,^9^ but there is no published information about parasitic fauna of cultured *A. persicus* in different growth stages. 

This study was conducted to investigate the parasitic fauna of cultured *A. persicus* (in propagation and rearing sturgeon centers) in different growth stages of life in northern of Iran. 

## Materials and Methods

This study was conducted in Shahid Rajaee Propagation and Rearing Center in the north of Iran. The experiments were conducted under identical conditions, following completely randomized design.^8^ A total of 5 samples from each sex breeders, 10 egg samples from female breeders, 20 samples from 5-day-old larvae, 80 samples from 20-day-old larvae and 60 samples from fingerling (that released in earthen ponds) were selected randomly. Samples were transferred by plastic bags contained oxygen to central laboratory of the Caspian Sea Institute, Sari, Iran.^8^

After the biometric measurements, wet mount from internal and external organs of larvae, skin, fins, gills and eyes of fingerling and breeders were prepared.^10^ After the preparation of wet smear, external parasites was identified by Bazari Moghaddam *et al.* key, using Klein's silver impregnation technique.^9^ In order to investigate gastro-intestinal parasites, macroscopic examination was done and then the entrails of the fish were removed. Also, approximately 50 to 60 eggs samples were collected from the ovary of female breeders and transferred to the central laboratory of the Caspian Sea Institute.

All collected parasites specimens were removed and stored in 70% alcohol. Then, cestodes, trematodes and acanthocephalans were stained with aqueous aceto-carmine and nematodes were cleared in lacto phenol.^11^ The specimens were identified by Bauer and Moravec keys.^12^^,^^13^

**Statistical** **analysis.** The data were subjected to statistical analysis using the SPSS (Version 18; SPSS Inc., Chicago, USA). Results of this study were analyzed statistically using One – way analysis of variance (ANOVA) and the significance level was expressed as *p *< 0.05. Also, Tukey’s test applied to compare between different groups. Mean intensity was determined by dividing the total number of recovered parasites to the number of infected fish samples. Prevalence was also calculated by dividing the number of infected fish samples by the total number of examined ones and expressed as a percentage. 

## Results

**Fig. 1:**
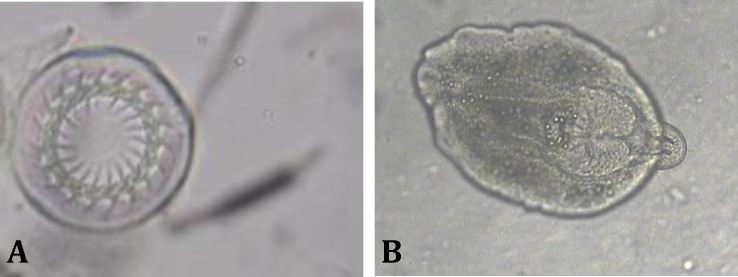
**A)** *Trichodina *sp. isolated from skin of 20 day larvae. **B)*** Diplostomum spathaceum* isolated from the eye of female breeder (100**×**).

According to the results, no parasite species were isolated from 5-day larvae with a mean weight of 54.6 ± 8.56 mg and mean length of 19.6 ± 0.96 mm (*p* > 0.05, r = 0.024), while protozoan *Trichodina *sp. was identified (Fig. 1A), from 20-day-old larvae with a mean weight of 78.50 ± 0.60 mg and mean length of 25.40 ± 0.51 mm (*p* > 0.05, r = 031). Mean intensity, prevalence and range of infection to *Trichodina *sp. were 2.50 ± 2.20, 13.90% and 1-3, respectively (Table 1). This protozoon was isolated from fingerling that released in earthen ponds (with a mean weight of 415.60 ± 39.67 mg and mean length of 40.60 ± 5.96 mm; *p* > 0.05, r = 035), the mean intensity, prevalence and range of infection fingerling were higher than 20-day-old larvae (*p* = 0.01, df = 2). In female breeders (with a mean weight of 31.14 ± 0.15 kg and mean length of 164.00 ± 0.96 cm; *p* > 0.05, r = 0.058), two parasites including: *Trichodina *sp. from skin and *D. spathaceum* from the eyes were isolated (Fig. 1B). Intensity, prevalence and range of infection of *Trichodina *sp. and *D. spathaceum* were calculated 8.12 ± 6.63, 40.00%, 1-4 and 1.26 ± 5.26, 20%, 1-6, respectively (*p* > 0.05, r = 0.034), (Table 1).

**Table 1:** External parasites in various stages of reproduction and rearing of *Acipenser persicus*. Data are presented as mean ± SD

**Sample**	**No.**	**Weigh**	**Total length**	**Parasites**	**Intensity**	**Prevalence (%)**	**Range**
**Female breeder**	5	31.14 ± 0.15 kg	164.00 ± 0.96 cm	*Trichodina *sp.	8.12 ± 6.63	20	1 - 4
				*I. multifiliis*	0	0	0
				*D. spathaceum*	1.26 ± 5.26	20	1 - 6
**Male breeder **	5	30.90 ± 0.14 kg	165.00 ± 0.19 cm	*Trichodina *sp.	11.76 ± 18.64	50	2 - 20
				*I. multifiliis*	138.50 ± 146.64	50	10 - 267
				*D. spathaceum*	0	0	0
**20 ** **day larvae**	80	78.50 ± 0.60 mg	25.40 ± 0.51 mm	*Trichodina *sp.	2.50 ± 2.20	13.90	1 - 3
				*I. multifiliis*	0	0	0
				*D. spathaceum*	0	0	0
**Fingerlings**	60	415.60 ± 39.67 mg	40.60 ± 5.96 mm	*Trichodina *sp.	9.56 ± 5.50	35.70	2 - 36
				*I. multifiliis*	0	0	0
				*D. spathaceum*	0	0	0

**Fig. 2.**
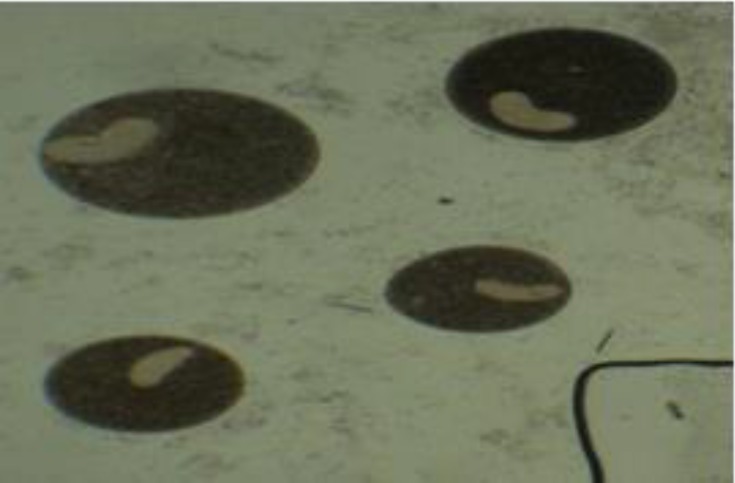
*Ichthyophthirius multifiliis* isolated from the skin of sturgeon fingerlings (40×).

Two protozoi including: *Trichodina* sp. and *I. multifiliis* were isolated from skin of male breeders with a mean weight of 30.90 ± 0.14 kg and mean length of 165.00 ± 0.19 cm (*p* > 0.05, r = 0.126), (Fig. 2, Table 1). Intensity, prevalence and range of infection in male breeders was estimated 11.76 ± 18.64, 50.00%, 2-20 and 138.50 ± 146.64, 50.00%, 10-267, respectively (*p* > 0.05,r = 0.051).

**Fig. 3:**
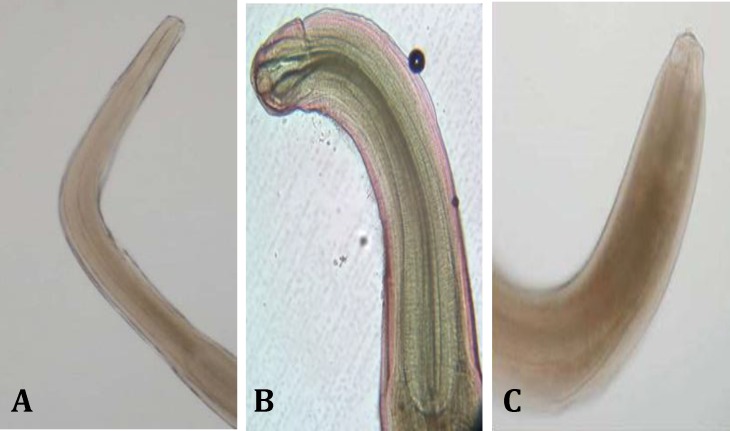
**A)** Anterior end of *Anisakis *sp. isolated from intestinal of male breeder (40×); **B)** *Cucullanus sphaerocephalus *isolated from intestinal of male breeder (100×); **C)** Posterior end of *Anisakis *sp. isolated from intestinal of male breeder (40×).

In this investigation, no parasites were detected from internal organs of larvaes and fingerling but four intestinal parasites, including:* Cucullanus sphaerocephlaus*, *Anisakis *sp.,* S. semiarmatus*, and *L. splagicephalu* were isolated from internal organs of the breeders (Figs. 3A and 3B, Tables 2).

**Table 2.** Internal parasites in various stages of reproduction and rearing of *Acipenser persicus *(n = 5). Data are presented as mean ± SD

**Sample**	**Weigh**	**Total length**	**Parasites**	**Intensity**	**Prevalence (%)**	**Range**
**Female breeder**	31.14 ± 0.15 kg	164.00 ± 0.96 cm	*C. * *sphaerocphlaus*	10.32 ± 18.50	40	3-17
			*L. * *plagicephalus*	5.62 ± 6.60	20	1-5
			*S.* *semiarmatus*	1.67 ± 1.15	20	1-2
			*Anisakis *sp.	1.23 ± 2.63	10	1-3
**Male breeder **	30.90 ± 0.14 kg	165.00 ± 0.19 cm	*C. * *sphaerocphlaus*	20.59 ± 32.5	40	15-25
			*L.* *plagicephalus*	1.78 ± 3.25	20	1-4
			*S.* *semiarmatus*	4.50 ± 1.50	40	3-6
			*Anisakis *sp.	0	0	0

In this survey, *Anisakis *sp. was isolated (Fig. 3) only from intestine of male breeds (10.00%), while others were found in both sex breeders (*p* < 0.05, r = 0.034). In intestinal infection, *Cucullanus sphaerocephlaus* had the highest intensity and frequency which, were observed in both examined sex breeders (*p* > 0.05, r = 0.124).

## Discussion

In recent years, due to losses in *A. persicus* stocks in the Caspian Sea, many researchers have paid attention to revive the sources of this valuable species.^1^ By increasing and developments in rearing of sturgeons in ponds of Iran, identification of pathogenic agents in order to apply the best methods of prevention and treatment seemed necessary. 

In this study, no parasites were isolated from 5-day-old larvae of *A. persicus*; but *Trichodina *sp. was isolated from 20-day-old larvae, these results were similar to Bazari Moghaddam *et al.* study. This finding may be related to the short larval rearing period in Vniro ponds (varied between 2-3 weeks), absence of intermediate hosts and also the lower water temperature in ponds compared to fingerlings in earthen ponds and lower organic load in Vniro ponds, presence of parasitic agents seemed lower than in rearing in earthen ponds.^9^^-^^11^

In Shahid Rajaee complex the fingerlings of *A. persicus *infection to *Trichodina *sp. (36.70%) were in line with our findings.^6^ However, in studies conducted by Ghoroghi the contamination percent of *A. persicus* fingerlings compared to *Trichodina *sp. was 22.00%, in our data showed lower numbers.^14^ Results obtained from the studies conducted by Shenavar Masouleh *et al.*, indicate that intensity and contamination range of *Trichodina reticulate* infestation in *A. persicus *fingerlings at the first week in earthen ponds were 20.00 to 30.00% and 2 to 10, respectively,^6^ that compared to our results was higher.

Similar to our results, in Shenavar Masouleh *et al.* study,* D. spathaceum* was not observed in the first week in earthen ponds. Limitation on the maintenance time in raising ponds and consequently the uncompleted life cycle of parasites, the presence of a small number of intermediate hosts, un-favorable temperature conditions for parasite growth are the possible reasons for the limited number of parasites during raising period of fingerlings in ponds.

This study showed the presence of four intestinal parasites, including: *L. plagicephalus*, *S. semiarmatus*, *C. sphaerocephlaus*, Anisakis sp*.* in *A. persicus *breeders. Ali Mohammadi *et al.* isolated the same parasites from *A. persicus* breeders on the southern coast of the Caspian Sea.^15^ Also, Sattari and Khara *et al.* not only reported these parasites, but also isolated *Eustrongylides excicus* in sturgeon’s species in southwest coast of Caspian Sea.^16^^,^^17^ Ghoroghi reported seven intestinal parasites in *A. persicus *and indicated that *C. sphaerocephlaus* and *S. semiarmatus* had the highest intensity and contamination rate.^14^ Also, Bazari Moghaddam found that *C. sphaerocephlaus *had the highest intensity and contamination rate between intestenial parasites in *A. persicus *breeders,^11^ these results was similar to our results and confirmed it. The increase in these parasite communities may be related to the occurrence of Nereids (polychaeta), the intermediate host for *C. sphaerocephalus.*^6^

In the present study, *Anisakis *sp*.* was only found in male breeders. However, Ali Mohammadi *et al.* reported this nematoda in both sex of breeders.^15^ Sattari and Sattari and Mokhayer isolated *Anisakis *sp. from other sturgeon species such as: *Huso huso* and *A. persicus*.^8^^,^^16^ By considering the high ability of this nematode to cause disease in humans and creating the allergic reactions, comprehensive studies on Anisakidea and other zoonotic parasites in sturgeons species seems to be essential.^18^^-^^20^

Due to important roles and negative effects of parasites infection in different growth stages of life in sturgeons and based on the results of previous studies,^15^^-^^18^ it seems following protocols should be observed; proper pond designing, controlling the water quality and quantity, using of disinfections, controlling of snails and aquatic birds as intermediate hosts, improving nutrition and decreasing stress condition in order to reduce the numbers of parasites infection and improvement on the health conditions of cultured sturgeons.^19^ Based on the results, periodic parasitical examination can contribute to the control of fish parasite and a reduction in the economic losses in propagation and rearing center of sturgeons species.
